# Significantly reducing the presurgical preparation time for anterior pelvic fracture surgery by faster creating patient-specific curved plates

**DOI:** 10.1186/s13018-023-03749-x

**Published:** 2023-04-01

**Authors:** Sendren Sheng-Dong Xu, Tsu-Te Yeh, Jia-En Chen, Yuan-Ta Li

**Affiliations:** 1 Graduate Institute of Automation and Control, National Taiwan University of Science and Technology, No. 43, Keelung Rd., Sec. 4, Da’an Dist., Taipei City, 106335 Taiwan; 2 Advanced Manufacturing Research Center, National Taiwan University of Science and Technology, No. 43, Keelung Rd., Sec. 4, Da’an Dist., Taipei City, 106335 Taiwan; 3 Department of Orthopedic Surgery, Tri-Service General Hospital, National Defense Medical Center, No. 325, Chenggong Rd., Sec. 2, Neihu Dist., Taipei City, 114202 Taiwan; 4 Medical 3D Printing Center, Tri-Service General Hospital, National Defense Medical Center, No. 325, Chenggong Rd., Sec. 2, Neihu Dist., Taipei City, 114202 Taiwan; 5 Department of Biomedical Engineering, National Defense Medical Center, No. 325, Chenggong Rd., Sec. 2, Neihu Dist., Taipei City, 114202 Taiwan; 6 Department of Surgery, Tri-Service General Hospital Penghu Branch, No. 90, Qianliao, Magong City, Penghu County 880026 Taiwan

**Keywords:** Computed tomography (CT)-based 3D medical imaging, Curved reconstruction plates, Anterior pelvic ring fracture surgery, Preoperative surgical planning, Three-dimensional (3D) printing

## Abstract

**Background:**

To shorten the preoperative preparation time, reconstruction plates were designed using the computed tomography (CT)-based three-dimensional (3D) medical imaging surgical planning software OOOPDS. In addition, 3D printing was used to generate curved plates for anterior pelvic fracture surgeries.

**Methods:**

This study analyzed two groups with the same 21 patients who underwent surgery for traumatic anterior pelvic ring fractures. In Group 1, the direct reconstruction plates were preoperatively contoured according to the anatomical 3D-printed pelvic model. In Group 2, the fixation plates were contoured according to the 3D printed plate templates, which were created based on the simulated plate templates by the OOOPDS software. The processing time, including the 3D printing time for the pelvic models in Group 1, the 3D printing time for the fixation plate templates in Group 2, and the pre-contouring time for the plates in both groups, was recorded.

**Results:**

The mean time of pre-contouring for the curved reconstruction plates in Group 2 was significantly less than in Group 1 (−55 min; *P* < 0.01). The mean time of 3D printing for the 3D plate template model in Group 2 was significantly less than that for the 3D pelvic model in Group 1 (−869 min; *P* < 0.01). Experimental results showed that the printing time for the plate pre-contouring and the 3D plate templates could be effectively reduced by approximately 93% and 90%, respectively.

**Conclusion:**

This method can shorten the preoperative preparation time significantly.

## Introduction

Pelvic ring injuries constitute only 2 to 8% of all fractures but occur in 20% of polytrauma patients, with a mortality rate of 10 to 16% [1]. Unstable pelvic fractures are often caused by a high-energy force, such as road traffic accidents, falls from a height, and localized crush injuries [2], and may result in uncontrolled hemorrhage leading to a high morbidity rate. It is a consensus that surgical intervention is required to treat unstable pelvic fractures. Pelvic fixation has traditionally been divided into posterior and anterior fixation. Although pelvic stability is mainly sustained by the posterior ring, the anterior ring provides 30% of the pelvic stability [3]. The fracture pattern dictates the type of implants and fixation strategy. Surgical intervention may be isolated to the posterior or combine posterior and anterior surgical fixations [4, 5]. Although stable and accurate posterior ring fixation remains important, the recognition that each injured pelvic ring element is a potential site of instability and deformity has driven the improved evaluation and fixation of the anterior pelvis.

Techniques for stabilizing the anterior pelvic ring include external fixation, symphyseal plating, intramedullary ramus screws, and an anterior subcutaneous internal fixator. Open reduction and internal fixations (ORIF) are associated with prolonged operative times and morbidities, while external fixators are associated with high complication rates. Plate fixation can be used for ramus fixation or to span both an injured ramus and symphysis pubis. In most instances, plate and screw fixation requires a formal open surgical approach to access the injured element(s) of the anterior ring.

The introduction of three-dimensional (3D) printing [6–22] to surgical planning has gained importance, considering the improvements in the accuracy and precision of surgical implant preparation, especially in pelvic surgeries. Pelvic fracture surgeries conventionally need complete access to the surgical site and intraoperative implant contouring following the fracture reduction [15, 16, 23–25]. Nevertheless, such an invasive approach invariably leads to prolonged surgeries and excess bleeding due to the complex regional anatomy and complicated fracture configurations [26]. Therefore, to present pelvic fracture patterns to surgeons for proper evaluation, 3D printing technology and virtual surgical simulation systems based on computed tomography (CT) have become essential methods for preoperative planning [15, 16, 27–31]. However, according to the study by Yeh et al. [16], printing life-size 3D pelvic models to shape customized implants is time-consuming.

In this study, a dedicated semi-automatic patient-specific reconstruction plate design system was used for anterior pelvic ring fracture surgeries combined with a newly introduced method using the OOOPDS software Version 1.06 (Taiwan Main Orthopaedic Biotechnology Co., Ltd; Taichung City, Taiwan) to create a curved plate from a series of selected visual points from the imaging. This study will provide a user-friendly interface for fracture reduction simulations and semi-automatic personalized plate design for fast preoperative implant templating. This study hypothesized that implant pre-contouring according to the 3D printed plate template model created based on the semi-automatic reconstruction system would be more efficient than plate pre-contouring according to the actual 3D printed pelvic model.

## Materials and methods

### Patient collection and image data acquisition

This retrospective study analyzed data from 21 patients with traumatic anterior pelvic ring fractures who underwent surgery at the Tri-Service General Hospital in Taiwan from November 2016 to February 2020. Pelvic CT images (3-mm axial slices) were obtained and saved in Digital Imaging and Communication in Medicine (DICOM) format. The image resolution ranged from 0.6 to 1.0 mm. Informed consent was obtained from all the patients, and the Institutional Review Board of the Tri-Service General Hospital approved this study.

### Segmentation and anatomical 3D virtual model reconstruction

Each patient’s pelvic thin-slice CT image was imported into the 3D medical image processing software (OOOPDS Version 1.06, Taiwan). The OOOPDS software’s volume-rendering technique was used to visualize the fracture, remove the surrounding tissues, and separate the structural parts from the soft tissue according to the variant threshold. After the bony structure was separated from the soft tissue, using the splitting process, the bilateral femurs were erased, and the pelvis was isolated. Different colors were assigned to the different fracture fragments, and each fracture fragment was divided into separate parts following the segmentation process. The software reduction techniques for fractured bones included segmentation, split, mirror, and manual repositioning techniques depending on the fracture patterns. After the 3D software processing, the simulated 3D pelvic model could be visualized from all directions during the virtual repositioning. Finally, the patient-specific anatomical reduction 3D pelvic model was reconstructed and saved in the stereolithography (STL) format.

### Selection of bone surface points for customized reconstructed fixation plate

**Fig. 1:**
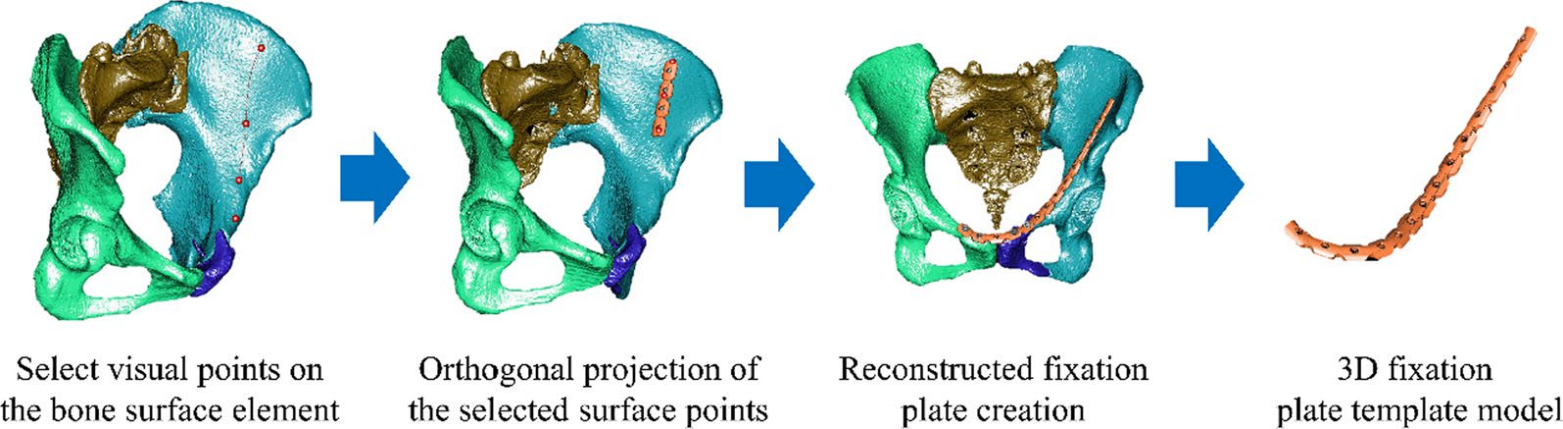
The semi-automatic patient-specific reconstruction plate design system

**Fig. 2:**
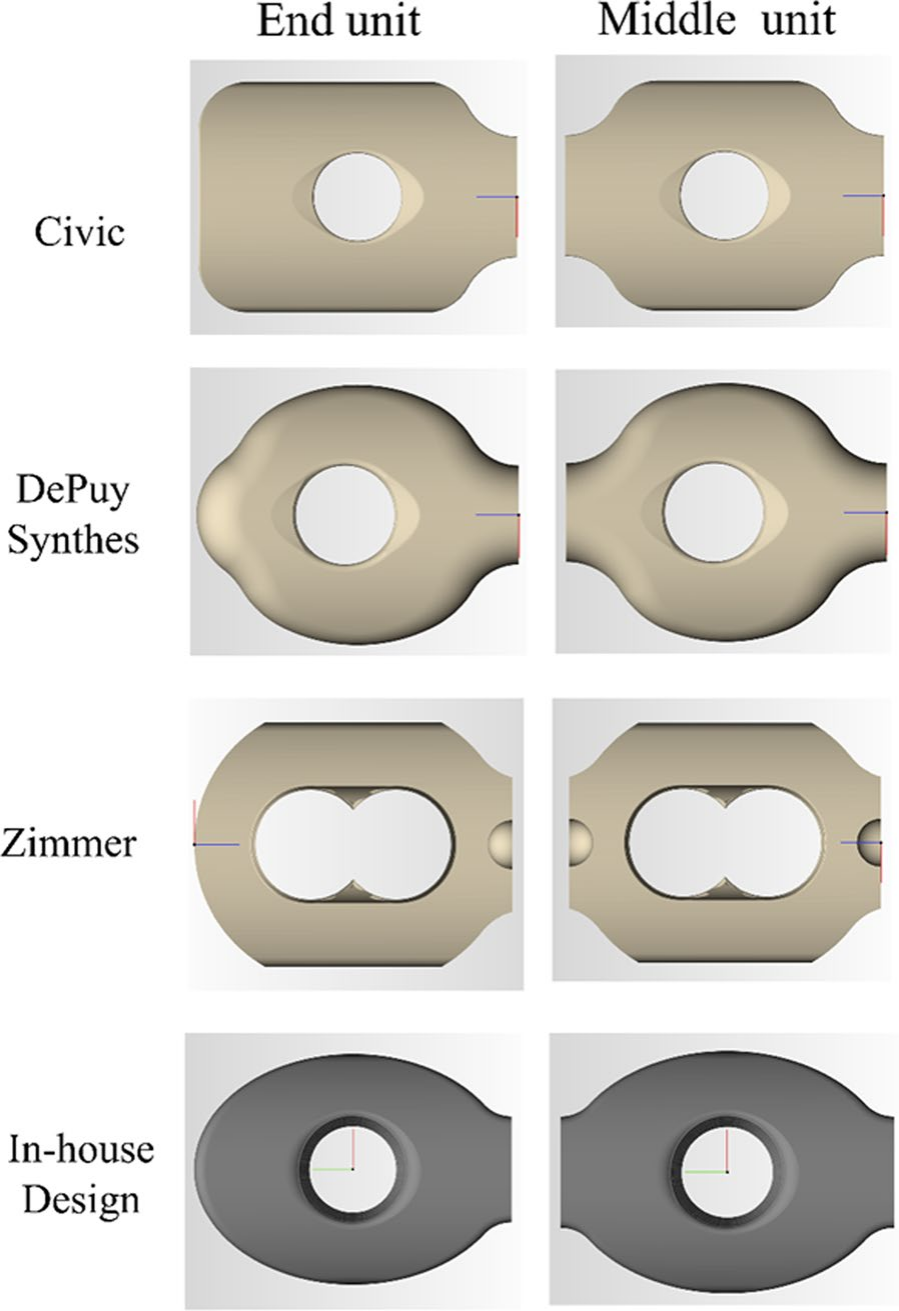
The 3D CAD models of each of the fixation plate units in the information library (Civic, DePuy Synthes, Zimmer, and In-house Design)

After the virtual reduction was completed, the curvature could be designed for ease of clinical use upon evaluating the position of the plate unit from a series of selected visual points (Fig. 1). Different fixation plates (Civic, Taiwan; DePuy Synthes, Switzerland; Zimmer, USA) could be chosen from the information library. An In-house Design unit was also created in the system for future studies. The orthopedic surgeon decided on the surface points on the pelvic models according to previous photographic records, and then the patient-specific plate template was generated automatically (Fig. 2).

### 3D printing technique

These patient-specific pelvic models (1:1 scale ratio) and plate templates were exported in the STL format and fabricated by a Fused Deposition Modeling (FDM) printer (Fortus 360, Stratasys, USA) with acrylonitrile butadiene styrene (ABS). The following print settings were used for all the 3D printing: travel speed 160 mm/s, print speed 65 mm/s, infill density 50%, layer height 0.2 mm, and support structures printed as lines with a thickness of 5% for structures with an overhang over 70°. The time of alignment, slicing, and printing was measured.

### Fixation plate pre-bending process

**Fig. 3:**
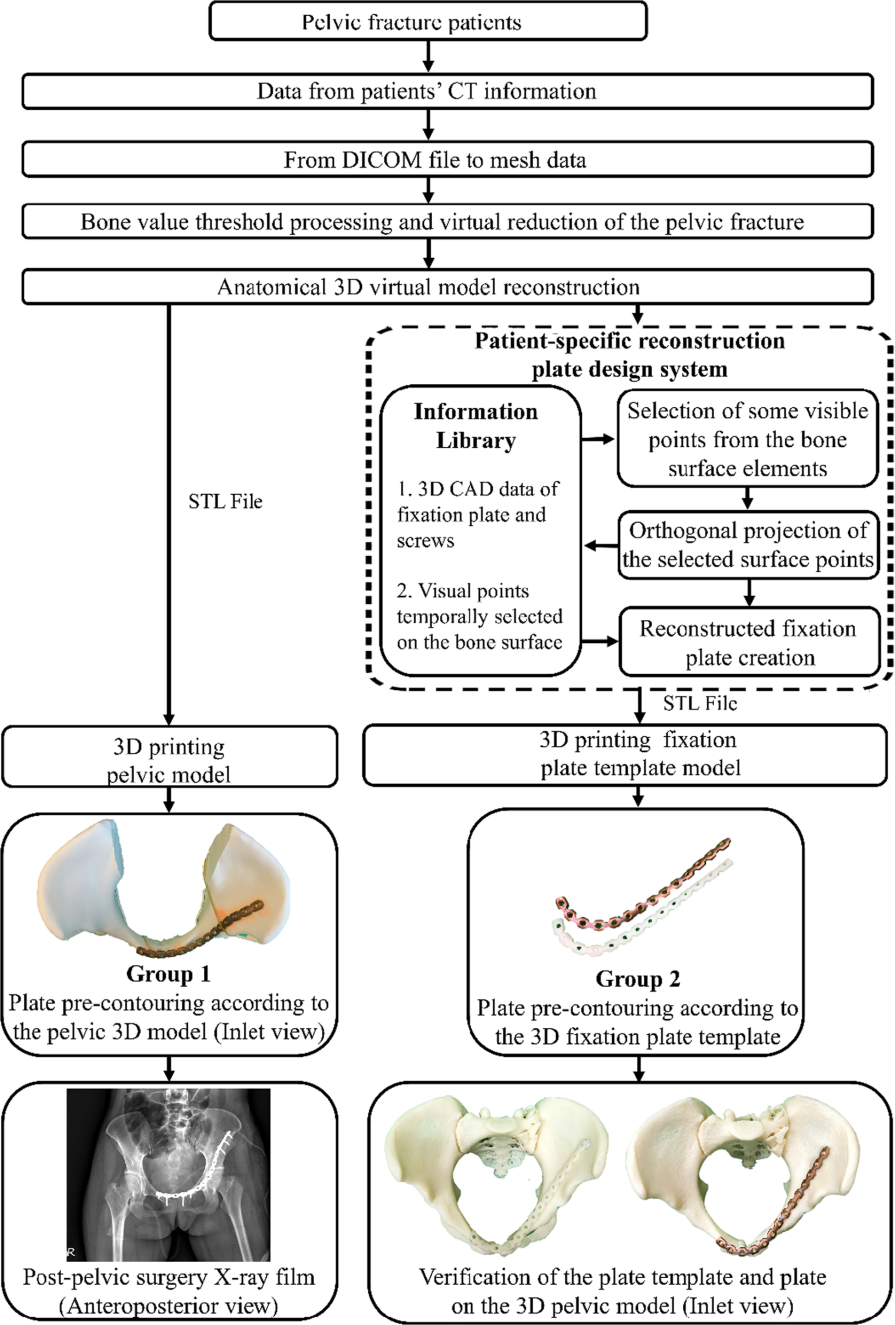
A flowchart of this study

This study used two groups to compare the plate pre-contouring time. Group 1 was composed of 21 patients with traumatic anterior pelvic ring fractures who had undergone surgery, and the straight reconstruction plate was contoured preoperatively according to the anatomical 3D pelvic model. The pre-contouring time of the plate before the surgery was recorded. In Group 2, using the same patients, the fixation plate template was generated using the semi-automatic plate generation system and 3D printing technique. The time spent shaping the straight reconstruction plate based on the 3D printed fixation plate template was recorded. The 3D-printing time for the pelvic models in Group 1 and the 3D-printing time for the plate template in Group 2 was also compared. All procedures were performed by the same orthopedic surgeon and surgical team. The flowchart of this study is shown in Fig. 3.

### Data analysis

Quantitative data are expressed as the mean ± standard deviation. Statistical analyses were performed using the Statistical Package for the Social Sciences software (version 22, SPSS, Inc., Chicago, IL, USA). A Wilcoxon signed-rank test was used in this study. A *P*-value of less than 0.05 was considered statistically significant.

## Results

**Fig. 4:**
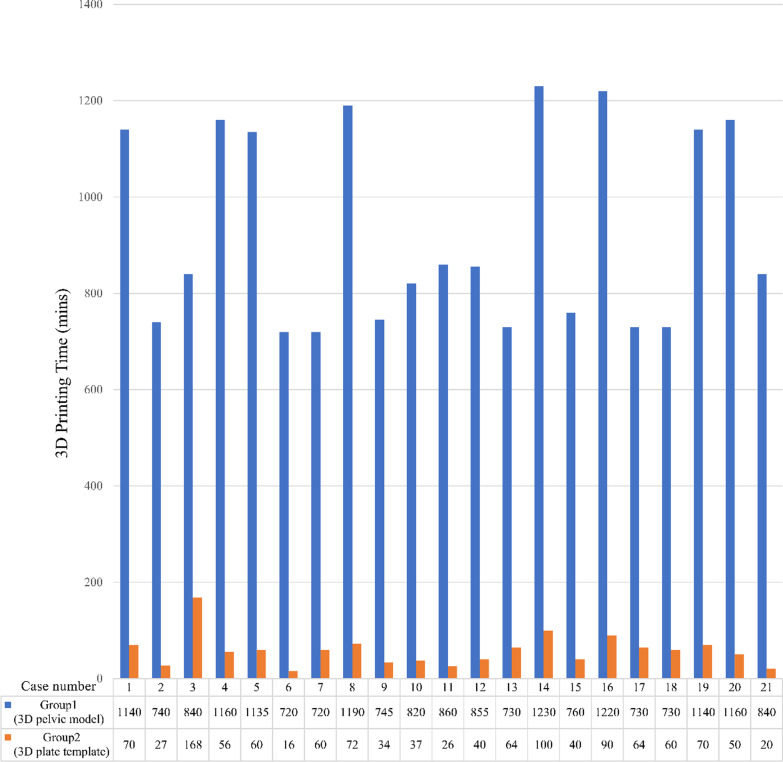
The calculated 3D printing time comparison between Group 1 (3D pelvic model) and Group 2 (3D plate template)

A total of 21 cases were included in this study. In Group 1, the mean printing time for the life-size anatomical pelvic 3D model was 926.9 ± 202.95 minutes. The mean time for the semi-automatic generation of the 3D plate template in Group 2 was 58.29 ± 33.45 minutes. The difference between the two groups was statistically significant (*P* < 0.01), and the average 3D-printing time was reduced by 90%. Individual data are shown in Fig. 4.

**Fig. 5:**
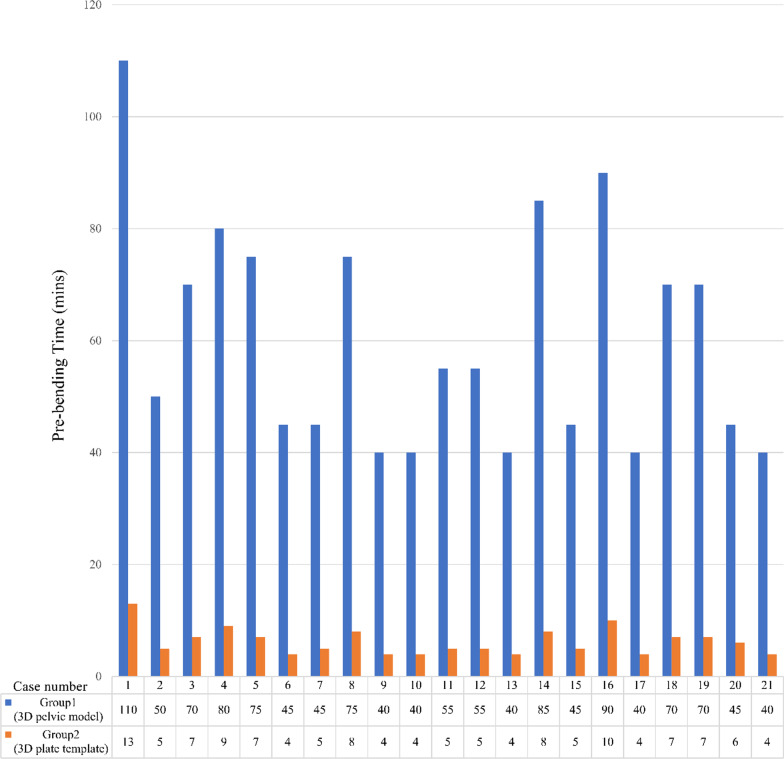
The comparison of the calculated plate pre-contouring time between Group 1 (3D pelvic model) and Group 2 (3D plate template)

The time required for preoperatively contouring the plate using the anatomical 3D pelvic model in Group 1 was 62.50 ± 21.45 minutes. In Group 2, contouring the plate using the semi-automatically generated plate template took 6.24 ± 2.39 minutes. There was a significant difference between the groups (*P* < 0.01), and the average pre-contouring time was reduced by 93%. Individual data are presented in Fig. 5.

## Discussion

This study showed that the time required for the 3D printing of the plate template model in Group 2 was significantly less than in Group 1 (*P* < 0.01). This finding may have resulted from the size of the 3D model. In Group 1, the life-size anatomical pelvic model was printed, whereas, in Group 2, only the plate template was printed. Hence, the printing time for the 3D plate template was reduced by approximately 90% by using the new method. Moreover, the mean pre-bending time in Group 2 was significantly less than in Group 1 (*P* < 0.01). This result implies that the surgical team contoured the fixation plate more efficiently, according to the 3D printed plate template than according to the 3D printed pelvic model. Thus, the pre-bending time was reduced by approximately 93% using the new semi-automatic plate design system. Compared to the whole pelvic model, the plate bending time was reduced significantly by using an isolated plate template model since the template provided precise information on the orientation and curvature of the plate. Additionally, the interference by the bony pelvic structure was avoided. Compared with Group 1, the reduction in time seen in Group 2 was due to forming the semi-automated plate template using the software. In this study, using this software, the average time for performing the semi-automated plate template was about ten minutes. In addition to the reduced time in Group 2, the cost was also reduced due to the different size of the model. Using this semi-automatic patient-specific reconstruction plate design system, the average ABS material cost for one 3D printing plate template (in Group 2) was about $1.50 US dollars. The average ABS material cost for one 3D printing whole pelvic model was about $220.00 US dollars due to the larger size of the model.

The software utilized a normal vector generated from the surface to ensure that each unit’s profile aligned parallel to the bone surface. Furthermore, algorithms were incorporated into the software to construct an optimal path. A threshold value was established in the generation formula to prevent the formation of a plate that would penetrate the surface. The smoothing function in the software was used to create smooth parts. A good fracture reduction was important for the virtual simulation in the software. When dealing with areas of poor continuity due to inadequate fracture reduction, the wrap function was employed. To ensure proper line length matching with a set of plate units, the line was cropped when the line was shorter than the mid-line of the fixation plate unit, and the line was lengthened when the line was longer than the mid-line of the fixation plate unit.

Pelvic ring fracture surgery is a challenging task for orthopedic surgeons. The surgery’s difficulties lie in the complex anatomy of the pelvis, the diversity of fracture morphology, and the inconvenience of contouring plates; thus, detailed preoperative planning combined with accurately contouring plates can ensure the success of pelvic ring fracture surgeries [30]. Moreover, it is essential to develop a preoperative surgical procedure that avoids suboptimal reductions [31]. Using virtual surgical planning tools with medical image data can facilitate accurate surgical planning and create a contoured plate appropriate for the patient’s anatomy [17]. Surgeons can determine the best sequential reduction procedure and choose the proper surgical approach by manipulating the segmented bones in a virtual simulation. In addition, fracture reduction can be more efficient during pelvic surgery when the pre-contoured plate serves as an anatomic plate; the concept is similar to using the anatomical locking plate for fracture fixation for the indirect reduction technique.

Making anatomical models using the patient’s medical imaging data with 3D printers is feasible [32]. The 3D printing method allows for the rapid construction of full-scale anatomical models to facilitate the visualization of intricate pelvic fracture patterns before surgery. More recently, studies have presented 3D printing technology and virtual planning techniques for pelvic ring fractures. These techniques can improve a surgeon’s efficiency, reduce iatrogenic complications, and shorten surgical time [17, 33]. To plan acetabular fracture surgery, Duncan et al. used a life-size model made by 3D printing technology and medical software, which converted DICOM data into an STL format [34]. Similarly, Brown et al. reported desirable results when using computer-generated 3D-moving representations to plan the placement of the plates to fix pelvic fractures [28, 35]. Maini et al. concluded that a patient-specific pre-contoured plate made using the 3D model was a better implant than an intraoperatively contoured plate [36]. Collectively, this growing body of literature demonstrates that 3D printing-assisted surgery has significant advantages in reducing the amount of intraoperative blood loss and shortening the duration of the surgery.

Chen et al. [33] indicated that all the preoperative preparations could take 36 hours. Therefore, 3D-model printing is the key to preoperative preparation time. Previously, solid pelvic models were printed to visualize the 3D structures to decide on the shape of customized implants and screw trajectories. With the novel technique provided by the OOOPDS software, the semi-automatic bone surface points used to generate the patient-specific plate templates significantly reduced the printing and pre-contouring times by 90 and 93%, respectively, in this study.

In bilateral pubic ramus fractures and unilateral pubic ramus fractures that occur near the pubic symphysis, the fixation plate should be placed bilaterally, and the length of the plate should be placed across the pubic symphysis to the contralateral side of the pubic bone. For these cases, a real-size pelvic 3D model should be printed. However, in unilateral pubic ramus fractures near the obturator foramen, the fixation plate should not be placed across the pubic symphysis. In this case, only one-half of a real-size pelvic 3D model should be sufficient for the pre-contouring of the plate, and the estimated printing time for the pelvic model could be reduced half of the time.

This study further pointed out that accurate bends and twists pose noticeable challenges in matching the plate to the template, although twists and gradual curves can mimic the use of manual plate benders. During the pre-contouring of the plate, the bending and curving of the straight plate should be performed between the adjacent screw holes; otherwise, the screw holes could be destroyed and make the part unsuitable for the surgery. Previous studies on the virtual creation of pelvic plate templates have focused solely on the curvature of the templates and have limited consideration of the real fixation plate configuration [23, 37]. In this study, different plates designed by various companies were created, and the selected surface points were produced to connect the plate units. This procedure was a simple method to overcome the problem of matching the plate to the template during pre-contouring.

This study defined a specific goal to successfully evaluate a technology that supports pelvic fracture treatments with the help of a semi-automatic patient-specific reconstruction plate design system and 3D printing. In addition, this study demonstrated the feasibility of a workflow incorporating a semi-automatic plate design system with the implant core unit library and 3D printed plate template technique for plate pre-contouring.

### Limitations

This study has some limitations. Since this study was retrospective, the pre-contoured plates in Group 1 had already been placed inside the patient. It was impossible to remove the implants and to do accurate tests with the pre-contoured plates in Group 1. In Group 2, the accuracy tests were performed by placing the pre-contoured plate on the patient’s 3D pelvic model for verification, shown in Fig. 3. All the pre-contoured plates fit the pelvic bone models, and the screw holes could be drilled into the model. In future studies, a prospective study could compare the pre-contoured plates prepared by these two methods by scanning the plates and plate templates to evaluate the fine differences by the software. Second, this study has a comparatively small sample size and did not follow a randomized-controlled and blinded study design. Therefore, a larger patient population is needed to assess its surgical applications further. Third, this study focused on the techniques and initial experiences rather than long-term clinical outcomes. Further studies should prospectively evaluate the effect of the 3D printing-assisted pre-contoured plate fixation methods on treatment outcomes.

## Conclusion

This study applied a hybrid scheme combining the curved reconstruction plate simulation and 3D printing technique for the fast construction of patient-specific implants for anterior pelvic fracture surgery. This method can significantly reduce the preoperative preparation time.

## Data Availability

The data which support the findings of this study are from the Tri-Service General Hospital. The data are not publicly available since they contain information that could compromise the privacy of the research participants.

## Abbreviations

3D: Three-dimensional
ABS: Acrylonitrile butadiene styrene
CAD: Computer-aided design
CT: Computed tomography
DICOM: Digital imaging and communication in medicine
STL: Stereolithography
